# Robotic hernia surgery IV. English version: Robotic parastomal hernia repair. Video report and preliminary results

**DOI:** 10.1007/s00104-022-01779-5

**Published:** 2022-12-08

**Authors:** Maxime Dewulf, Ulrich A. Dietz, Agneta Montgomery, Eric M. Pauli, Matthew N. Marturano, Sullivan A. Ayuso, Vedra A. Augenstein, Jan R. Lambrecht, Gernot Köhler, Nicola Keller, Armin Wiegering, Filip Muysoms

**Affiliations:** 1 Department of Surgery, Maastricht University Medical Center, Maastricht, The Netherlands; 2 Department of Visceral, Vascular and Thoracic Surgery, Cantonal Hospital Olten, Olten, Switzerland; 3 Department of Surgery, Skane University Hospital, Malmö, Sweden; 4 Department of Surgery, Division of Minimally Invasive and Bariatric, PennState Hershey Medical Center, Hershey, PA USA; 5 Division of Gastrointestinal and Minimally Invasive Surgery, Department of Surgery, Carolinas Medical Center, Charlotte, NC USA; 6 Department of Surgery, Sykehuset Innlandet Hospital Trust, Brumunddal, Norway; 7 Department of Surgery, Ordensklinikum Linz, Linz, Austria; 8 Department of Urology, Cantonal Hospital St. Gallen, St. Gallen, Switzerland; 9 Department of General, Visceral, Transplant, Vascular and Pediatric Surgery, University Hospital Wuerzburg, Oberduer. Str. 6, 97080 Wuerzburg, Germany; 10 Department of Surgery, Maria Middelares Hospital, Buitenring Sint-Denijs 30, 9000 Ghent, Belgium

**Keywords:** Parastomal hernia, Ileal conduit, Pauli procedure, Funnel mesh (IPST), Modified Sugarbaker technique, Parastomale Hernie, Ileum-Conduit, Pauli-Verfahren, Trichternetz (IPST), Modifizierte Sugarbaker-Technik

## Abstract

The surgical treatment of parastomal hernias is considered complex and is known to be prone to complications. Traditionally, this condition was treated using relocation techniques or local suture repairs. Since then, several mesh-based techniques have been proposed and are nowadays used in minimally invasive surgery. Since the introduction of robot-assisted surgery to the field of abdominal wall surgery, several adaptations to these techniques have been made, which may significantly improve patient outcomes. In this contribution, we provide an overview of available techniques in robot-assisted parastomal hernia repair. Technical considerations and preliminary results of robot-assisted modified Sugarbaker repair, robot-assisted Pauli technique, and minimally invasive use of a funnel-shaped mesh in the treatment of parastomal hernias are presented. Furthermore, challenges in robot-assisted ileal conduit parastomal hernia repair are discussed. These techniques are illustrated by photographic and video material. Besides providing a comprehensive overview of robot-assisted parastomal hernia repair, this article focuses on the specific advantages of robot-assisted techniques in the treatment of this condition.

**Video online:**

The online version of this article contains 4 videos. The article and the videos are online available (10.1007/s00104-022-01779-5). The videos can be found in the article back matter as “Electronic Supplementary Material”.

## Abstract (de)

Die chirurgische Behandlung parastomaler Hernien gilt als komplex und ist bekanntermaßen komplikationsträchtig. In der Vergangenheit wurden diese Hernien durch die Relokation des Stomas oder Nahtverfahren der Austrittstelle versorgt. In den letzten Jahren wurden verschiedene netzbasierte Techniken vorgeschlagen, die heute in der minimal-invasiven Chirurgie eingesetzt werden. Mit der Verbreitung der roboterassistierten Hernienchirurgie wurden die Netzverfahren weiterentwickelt und die Ergebnisse für die Patienten erheblich verbessert. In diesem Beitrag wird ein Überblick über die verfügbaren Techniken der roboterassistierten Versorgung parastomaler Hernien präsentiert. Es werden technische Überlegungen und erste Ergebnisse des roboterassistierten modifizierten Sugarbaker-Verfahrens, der roboterassistierten Pauli-Technik und der Verwendung des trichterförmigen Netzes IPST vorgestellt. Darüber hinaus werden die Herausforderungen bei der roboterassistierten Versorgung parastomaler Hernien am Ileum-Conduit diskutiert. Die Operationstechniken werden durch Foto- und Videomaterial veranschaulicht.

Those who treat parastomal hernias will find an ingenious description of their own experience in modern fiction: the beginning of Michael Ende’s novel, *The Neverending Story* (1979) [1]:
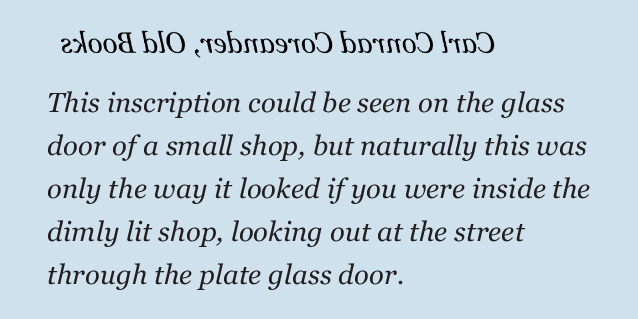


After what we have learned in the past 20 years with all the advancements in the surgical treatment of parastomal hernias, the impression arises that one only has to give a new technique enough time for the first recurrences and complications to occur. And after having been disappointed by an initially hopeful technique, one again sets off in search of new alternatives through the plate glass door of negative evidence back to open a path for another search, a new try, a new beginning. And in retrospect, one wonders about the obvious incoherencies of a once convincing concept. Aware of this repetitive history, we nevertheless want to present the current state-of-the-art of robotic repair of parastomal hernias. We are convinced that the robotic approach, supported by our matured experience with meshes and the recent improvements in knowledge of the anatomy of the abdominal wall planes, is the best that can currently be realized [2].

## Life with a stoma

One of the most prominent surgeons in history with a stoma was Edoardo Bassini (1844–1924), the creator of the first modern groin hernia repair technique; Bassini was wounded in 1867 with a bayonet as a volunteer in the army of Giuseppe Garibaldi to make Rome the capital city of Italy. As a consequence, he developed a cecal fistula, at his time called “anus praeternaturalis”; it is assumed that he worked his whole life as a surgeon with this posttraumatic stoma. Whether the fact that he had a stoma isolated him socially and whether the stoma was the reason that he was not married remains speculation. Life with a stoma is often associated with psychosocial problems and may seriously affect quality of life (QoL). Patients need to adhere to a different lifestyle when having a stoma. Around 0.45% of the population in the western world lives with a stoma, of which 60% are permanent [3]. The most common type of stoma is a colostomy (> 75% of all stomas being constructed). An ileostomy is commonly used for small bowel drainage or as a conduit for urinary drainage after bladder removal.

Proper stoma function is essential for good QoL. The function is dependent on the stoma type, stoma site, and the technical construction at the index operation. There is usually a team backing up the stoma care for patients, including a skilled colorectal surgeon or a urologist, who work in close collaboration with stoma care nurses. Patients receiving a stoma and their close family members will need significant amounts of information and psychosocial support in adopting a new lifestyle, both from a physical and psychological perspective. There are three validated instruments that could be used to describe QoL in stoma patients: EORTC (European Organization for Research and Treatment of Cancer) C30/CR38, MCOHQOLQO (modified City of Hope Quality of Life. Questionnaire Ostomy), Stoma QOL Questionnaire [4]. Studies have demonstrated that living with a colostomy has a negative impact on QoL. Problems and symptoms described were depression, constipation, gas, sexual discomfort, stoma appearance, clothing issues, feeling tired, fear of traveling, and concerns about noise.

Stoma-related problems are common, including bandage problems, skin erosions, and parastomal hernia (PH) formation. In a recent meta-analysis from 2022 involving over 1000 patients, stoma-related complications ranged between 3 and 80% [5]. Sore skin and ulcerations are the most common complications with an incidence of 25–35%. Another common problem is the development of either a subcutaneous siphon of the stoma, or the development of a “true” PH, where parts of intra-abdominal content herniate through the stoma aperture next to the stomal loop. The PH incidence is estimated to be around 30% at 12 months, 40% after 2 years, and > 50% during longer follow-up [6].

Technical considerations when constructing a stoma are vital for stoma function. A stoma needs maximal abdominal wall muscle support to minimize the risk of developing a parastomal hernia or a prolapse. The positioning of the stoma should be thoroughly discussed with the patient, giving pros and cons for different locations, in relation to the type of stoma. Skin quality, ease of handling stoma bandage exchange and patient’s preference of location in relation to clothing are to be discussed, as well as potential complications and problems that can arise when having a stoma. It is a delicate task to construct a well-functioning stoma that results in sustained quality of life. It is of the utmost importance to offer easy access, continuous and competent communication and support to all stoma patients. Special difficulties arise when a stoma has to be established in an emergency: no preoperative planning of the localization of the stoma on the skin, short, thick or inflamed mesentery, thick subcutaneous tissue or reduced perfusion of the intestine, just to name a few. Therefore, older patients who have undergone emergency stoma procedures are less likely to have their stoma closed, and often keep their stoma so that they do not have to undergo another operation.

## Parastomal hernia

A parastomal hernia (PH) can be defined as “an abnormal protrusion of the contents of the abdominal cavity through the abdominal wall defect created during placement of a colostomy, ileostomy or ileal conduit stoma” [7]. Generally, the surgical treatment of a PH is complex, and has proven to be prone to complications [8, 9]. As the local treatment of these hernias involves extensive dissection of the stomal loop and mesh placement in close relation to colon or small bowel, surgeons are often reluctant to perform this type of surgery. Besides relocation of the stoma, several techniques for the local repair of a PH have been proposed. Currently available literature advocates the use of a mesh when treating these hernias, and suture repairs are considered obsolete. Traditionally, a keyhole (where the stomal loop runs through the mesh) or the modified Sugarbaker repair (in which the stomal loop is lateralized and an intraperitoneal mesh is placed to cover the PH site) were used to treat these hernias. Several preshaped meshes have been evaluated in the surgical treatment of PH, which are mostly used in an intraperitoneal position [10]. More recently, Eric Pauli described a modification of the Sugarbaker technique, in which the stomal loop is lateralized in the retromuscular plane, and an extraperitoneal mesh is used [11].

In recent years, robotic-assisted surgery has been rapidly gaining popularity in abdominal wall surgery. For the treatment of PHs, it offers some specific advantages that could significantly improve patient outcomes. It enables

- Extensive adhesiolysis of the small bowel, from the duodenojejunal angle to the ileocecal region,
- Avoids the use of painful penetrating fixation techniques (e.g., tacks) during intraperitoneal repairs by facilitated suture fixation, and
- Allows the use of novel techniques using component separation techniques in a minimally invasive approach.

In this contribution, we aim to provide an overview of currently available robotic techniques in the repair of PHs, with an emphasis on the technical considerations. Furthermore, we aim to report on preliminary results of these techniques. This article is the fourth in a series of technical notes on the use of robotic hernia surgery [12–14].

## Indications for robotic parastomal hernia repair

Stomas may have different anatomical characteristics: end-colostomy, loop-colostomy, end-ileostomy (as enterostomy or ileal conduit), or loop-ileostomy. The site of the stoma may vary in relation to the topography of musculoskeletal structures (e.g., transrectal, lateral to the rectus muscle, close or far from bony structures). The degree of eversion of the intestine at skin level may also play an important role in its function and impact on quality of life. In general, the indication for PH repair is considered justified if there are skin-care problems, if the stoma bag leaks, the patient has pain or bowel obstruction symptoms. Furthermore, prolapse of the stoma with or without bleeding of the mucosa or polyps may be indications for repair. In general, there is no good way to repair a PH of a loop stoma, a rare situation that may complicate the treatment of patients with, for example, severe dysmotility disorders.

Preoperatively, computed tomography (CT) imaging of the abdomen and pelvis is typically obtained, which allows for elucidation of hernia characteristics, such as defect width, other hernias, incarcerated intestine, siphon-type adhesions of the bowel in the hernia sac, previously placed meshes, and tacks in the case of recurrent hernias. Having images available allows for appropriate operative planning and helps the operative surgeon to determine important steps of the operation, such as the appropriate location of entry into the abdominal cavity. Colonoscopic screening as part of cancer surveillance should also be considered. There should be a conversation between the surgeons involved in the patient’s care (e.g., colorectal surgery, urology) to determine whether patients are eligible for reversal of their stoma. If this is the case, stomal reversal should be performed instead of PH repair. Like patients with other types of ventral and incisional hernias, medical comorbidities are optimized before the operation [15, 16]. A body mass index (BMI) of less than 35 kg/m^2^ is generally strived for.

The inability or unwillingness to properly optimize patients is surprisingly common and can have a devastating impact on the wound complication rate, which can predispose patients to hernia failure or even mesh infection [15, 17]. The primary goal of PH repair is to create a durable repair and improve patient’s quality of life. All efforts should be made to avoid complications that can precipitate the need for reintervention, worsen the patient’s quality of life and generate a significant cost to the patient and the healthcare system [18].

## Robotic approach to parastomal hernia

In the operating room, the patient is induced under general anesthesia and laid supine. The patient is positioned to edge of the operating table at the ipsilateral side of the planned trocar position, to optimize free movement of robotic arms; the arm that is ipsilateral to the trocars and contralateral to the stoma is tucked and positioned slightly below the level of the core. The table may be flexed, which assures optimal exposure of the abdomen and allows for increased working space for robotic instruments. When a bilateral transversus abdominis release (TAR) is required, ports will be placed at both sides. Patients have padding placed at pressure points to prevent neurovascular injuries from developing during the operation. A urinary catheter is placed for patients with colostomies or ileostomies, and a sterile Foley catheter is inserted into ileal conduits to allow for drainage and identification of the conduit. The stomal opening may be closed with suture and prepped, then an occlusive dressing is placed over the stoma during surgery to minimize spillage and prevent contamination on the wound. Prophylactic antibiotics, typically a first-generation cephalosporin, may be administered, according to local hospital protocols. Patients are prepped and draped in a sterile fashion ensuring that draping exposes the abdominal wall far laterally on both sides of the abdomen. The DaVinci Xi (Intuitive Surgical, Sunnyvale, CA, USA) with double console and table motion (Trumpf Medical, Germany) is our preferred option.

Independent of the type of robotic PH repair, the positioning of the ports should be a compromise between optimal distance to the hernia site, considering the adjacent bony structures and the presence of adhesions. Adhesiolysis is often needed and care must be taken to recognize an enterotomy made during the dissection. At any point during the operation if the dissection becomes too difficult to carry out robotically or the patient is unstable, the decision should be made to work in hybrid fashion (e.g., open peristomal adhesiolysis) or convert to an open operation. If there is concern about the blood supply to the mesentery, then 0.2 mg/kg per 3 mL (approximately 5 mg in 1 mL suspension) of indocyanine green (ICG) can be injected intravenously, and the robotic Firefly technology can be utilized to evaluate perfusion [19].

## Modified Sugarbaker technique

**Fig. 1:**
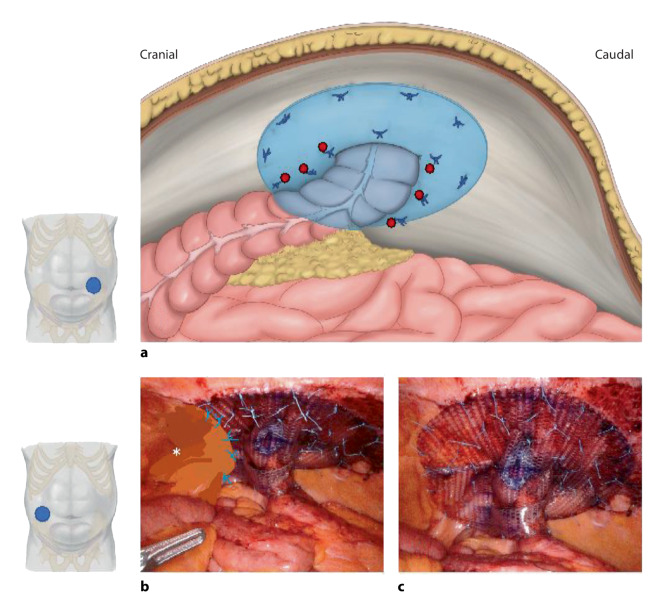
Robotic parastomal hernia repair with modified Sugarbaker technique. **a** Overview of the repair of an end-colostomy; suture mesh fixation (*red dots*) with creation of a lateralized tunnel; **b** aspect of an end ileostomy, the distal part of the mesh was extraperitonealized (*asterisk*); **c** ileostomy, in this case covering another former keyhole mesh (recurrence treatment). (**a** is adapted from Dietz et al. [21])

The first technique for PH repair to be discussed is the robotic modified Sugarbaker technique. Originally described to be performed by laparotomy, the Sugarbaker technique aimed to close the hernia orifice with an intraperitoneally placed prosthetic mesh that was sutured to the fascial edge, without mesh overlap. Later the technique was adapted to laparoscopy and modified in such a way that the mesh size was enlarged to allow a circumferential overlap of 3–5 cm with the mesh positioned in a typical intraperitoneal onlay mesh (IPOM) position (Fig. 1a; Video 1 [8]). The robotic modified Sugarbaker technique is ergonomically more favorable than the laparoscopic approach as it allows for easier visualization, dissection, and suturing. Using robotics, fascial closure is fairly simple [20].

### Technique

The operation begins with entry into the abdomen, which is most often performed using an 8‑mm trocar in the upper quadrant that is opposite the stoma. Two more ports are placed through the transversus abdominis muscles inferiorly to the initial port and on the same side of the abdomen. The one in the mid-abdomen is a 12-mm port and is used for the camera and mesh placement, and the more inferior port is an 8‑mm working port. These ports are placed at least a handbreadth apart to optimize the working space. Sutures and the mesh may be inserted into the abdominal cavity at this procedure. Mesh is chosen and sized according to size of hernia(s) to assure sufficient overlap. Synthetic mesh is avoided in contaminated cases. Several 0 nonabsorbable, braided sutures are tied to the mesh in the center and other directions to maintain orientation and provide initial apposition to the abdominal wall. The mesh is rolled up and placed through the 12-mm port. Most commonly, a midweight dual-coated polytetrafluoroethylene (PTFE) is used, as it is smooth and has a low risk of erosion into the bowel. Following port placement and insertion of the mesh, the Da Vinci robot is docked.

The camera is inserted into the 12-mm port, and then a nontraumatic grasper is inserted into the left hand and a monopolar scissor into the right hand under direct supervision. Meticulous adhesiolysis is carried out with blunt and sharp dissection, and the hernia sac is reduced intra-abdominally. In order to ensure maximal mesh overlap, a preperitoneal flap is then created in the inferolateral position starting at the site of the fascial defect. This flap is similar to the later dissection performed for a minimally invasive inguinal hernia repair and will allow for large mesh overlap inferiorly (preperitoneal plane; Fig. 1b). The peritoneum is eventually used to cover part of the mesh and care is taken to avoid placing tacks too far inferiorly towards the inguinal canal, which could result in injury to neurovascular structures. The fascial defect is usually closed with a nonabsorbable 0 UPS barbed suture, either as a purse string suture or as a running suture starting at one of the edges, so that the fascia is snug around the intestine, but not too tight. The intestine, along with its corresponding mesentery, is secured laterally to the abdominal wall to create a lateral tunnel for the modified Sugarbaker repair.

The mesh is then secured to the anterior abdominal wall using the preplaced stitches (transfascial or robotic suture on the abdominal fascia), all of which are tied down in an interrupted fashion. Placing the central suture at the most proximal edge of the fascial defect allows for the mesh to be centered and mesh suspended; this in turn aids in positioning and fixation of the rest of the mesh. A Sugarbaker tunnel is built with the mesh cradling the intestine to the abdominal wall (Fig. 1a, *red dots*). Lateral sutures are placed at each edge of the tunnel. One must take care not to tighten the mesh here, as it may result in bowel obstruction. When the lateral stitch is anchored to the abdominal wall, care needs to be taken to not pass the suture through the intestine or mesentery. The sutures on the periphery of the mesh may then run circumferentially to ensure that that the mesh is taut against the abdominal wall (Fig. 1c). The peritoneal pocket that was created in the inferolateral position is secured over the mesh with care to place the sutures and avoid neurovascular or bladder injury (Fig. 1b). All needles are removed and prior to desufflation, the operative site is re-examined to ensure that there is no bleeding or injury to the bowel.

Pitfalls may be inadequate mesh overlap, and securing mesh too tightly to the abdominal wall. When there is redundancy of the bowel or mesentery, it has the potential to re-herniate underneath the mesh and lead to incarceration.

### Results and comments

The results of this technique were recently published [20]. In this high-risk subset of 15 patients, almost all patients had their ostomies created for oncologic purposes, and 60% of whom were smokers but quit smoking at least 4 weeks prior to surgery. One enterotomy was made during dissection and a biologic mesh was used for that patient. The median length of stay was 2 days which is significantly shorter than open and laparoscopic PH-repair patients [22, 23]. The mean operative time was 3 h. Postoperatively, there were no wound complications or 30-day readmissions. One patient (6.7%) developed a hernia recurrence during mean follow-up of 14.2 (±9.4) months. The patient who developed a recurrence was morbidly obese (BMI 38) and had multiple prior PH repairs with mesh. This patient was also one of the first cases performed and influenced the development of the peritoneal flap to assure more extensive inferior mesh overlap.

## Pauli technique

In 2016, Pauli et al. described a retromuscular parastomal hernia repair method combining transversus abdominis release (TAR) with the advantages of the tunnel of the Sugarbaker configuration with extraperitonealization of the mesh (Video 2; [11]). Although originally described as an open operation, laparoscopic and robotic versions of the surgery have now been reported [24, 25].

The primary concerns with the standard intraperitoneal non-slit mesh repair are mesh complications and acute and chronic pain from mesh fixation. In addition, the recurrence rates continue to be significant, and repair is not risk-free [26]. The Pauli technique was developed to decrease recurrence rates compared to keyhole retromuscular techniques, decrease surgical site occurrences compared to relocation techniques—and to keep mesh away from the abdominal cavity [11, 27].

### Technique

Most often a “transabdominal” approach is employed for safer reduction of the hernia content. Entrance into the retrorectus space from the abdominal cavity through the peritoneum and posterior rectus sheath is adjacent to linea alba on the ipsilateral side of the stoma. If the medial distance from the ostomy to the linea alba is insufficient for adequate overlap of the reinforcing mesh, a truncated TARUP (transabdominal retrorectus umbilical prosthesis) technique with midline crossover behind the linea alba can be used [13, 28]. The procedure can also be performed extra peritoneally (extended totally extraperitoneal prosthesis, eTEP) with direct entrance to the retrorectus space, with or without preperitoneal cross-over (crossing under the linea alba) of the midline. For concomitant midline hernia repair an eTEP entrance contralateral to the stoma with midline crossover can be applied—and in need of bilateral TAR, redocking to opposite-side trocars is typically needed both with eTEP access and transabdominal access, as described for the robotic TAR [14].

**Fig. 2:**
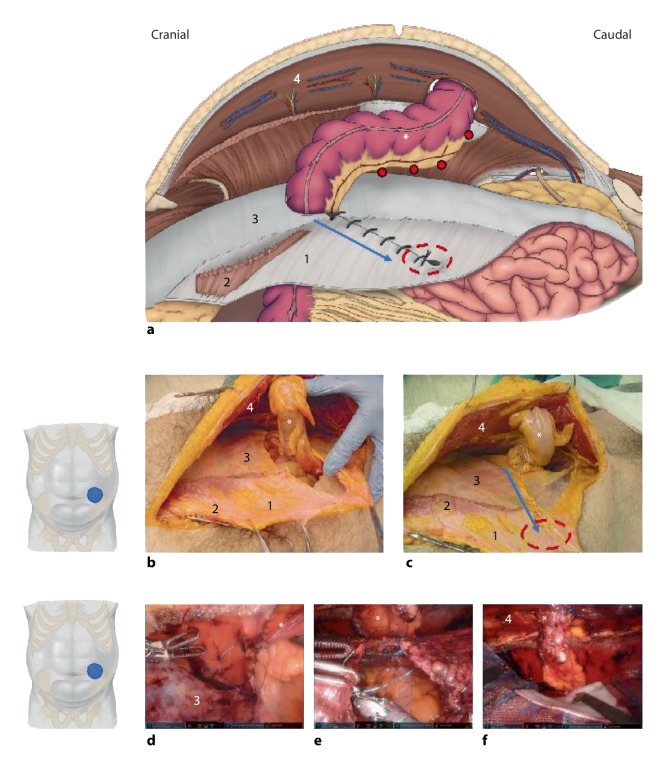
Robotic parastomal hernia repair with the Pauli procedure. **a** The layers of the abdominal wall at the side of the parastomal hernia are separated as described for the transversus abdominis release (TAR) technique [14]; the bowel (*asterisk*) is lateralized and suture-fixed to the abdominal wall (*red dots*); the original opening of the stoma is closed at level of the posterior rectus sheath (*red dotted circle*); after lateralization of the opening orifice the posterior rectus sheath is closed from lateral to medial (*blue arrow*); **b,c** cadaveric dissections of the relevant anatomy. **d** Suture fixation of the bowel to the abdominal wall. **e** Suture closure of the posterior rectus sheath from lateral to medial, beginning laterally at the new opening for the transit of the bowel. **f** Placement of the mesh and protective scaffold. *1* posterior rectus sheath, *2* released medial insertion of the transverse muscle, *3* endoabdominal fascia, *4* rectus muscle

After dissection and exposure of the retrorectus space at least 10 cm cranially and caudally to the ostomy, the hernia sac is incised circumferentially. Then a TAR dissection is launched lateral to the stoma. A TAR can be started cranially, or caudally from the Bogros space. Starting from Bogros space, the plane developed is typically posterior to the transversalis fascia. The peritoneum is very thin in the cranial part of the anterior abdominal wall. The posterior fascia and the transverse muscle are incised medially to the neurovascular bundles and the space is developed in the lateral direction. The lateral dissection in the TAR plane is continued at least 10 cm lateral to the hernia defect. After the development of the TAR plane and a landing zone for a mesh, preferably not less than 18 cm in craniocaudal length, the posterior retromuscular fascia is incised lateral to the stoma all the way to the edge of the developed TAR plane (Fig. 2a). Next, the stoma is repositioned by internal traction and external manipulation in anticipation of a post-hernia position and trajectory, to have a bowel course through the abdominal wall and the mesh funnel without folds (Fig. 2a–c).

The bowel is fixed to the anterior abdominal wall with typically a 23 cm long 2‑0 absorbable barbed running suture, grasping the mesentery with care not to violate the vascular supply, from lateral to medial for 6–7 cm at the cranial side (Fig. 2d). Care must be taken to avoid retraction of the stoma at the skin level and at the same time to avoid a convoluted passage through the abdominal wall. Suturing the bowel to the abdominal wall, prevents dislocation of the bowel and mesh fixation is superfluous in most cases. On the caudal border of the stoma loop, the mesentery is only fixed laterally before the dorsal fascia is closed by running barbed suture from lateral to medial (Fig. 2a,c: *blue arrow*; Fig. 2e). Care must be taken not to strangulate the bowel with a too tight internal opening to the peritoneal cavity, but still a firm closure must be obtained. The bowel is thus secured, and a retromuscular tunnel has been created with lateralization of the inner ostomy. The aim is a distance of at least 8 cm from the lateral edge of the outer ostomy. The outer ostomy with the hernia defect is then partially closed and adapted to the passing bowel.

A ruler is used to gauge the space for mesh placement. The size of the mesh should be a minimum of 18 cm by 18 cm. The mesh used should be large-pore, synthetic and nonabsorbable of polypropylene, or polyester material. However, concerns with mesh erosion into the intestine and mesentery with late complications as devascularization, infection, and fistula formation may warrant a protective cushion between the nonabsorbable mesh and the bowel (Fig. 2f). Utilizing a preproduced barrier mesh or fashioning a separate barrier mesh insert can be considered. In high-risk fistula cases as with Crohn’s disease, there is no safe solution but using an absorbable fibrin-scaffolding barrier mesh (e.g., a biosynthetic absorbable mesh) or maybe a large-pore polytetrafluoroethylene (PTFE) material could be favorable. Most often the mesh placement is secure from migration without fixation, but the mesh can be suture-anchored to the anterior abdominal wall if difficult to keep in position before desufflation. The alleged advantages with bowel fixation and no mesh fixation are that the bowel is secured against migration during healing and the mesh can adapt to the bowel, conceivably reducing the risk of bowel strangulation at the mesh edge. Finally, the retromuscular pocket is closed by suturing the posterior fascia to the linea alba in case of a transabdominal access without midline crossover.

### Results and comments

Adopting the Pauli principle to endoscopic surgery, we have experience from 26 robotic Pauli procedures, with a follow-up of median 14 months (range 0–30 months). Our patients had a mean age of 64 ± 8 years, 14 (54%) were males, the median BMI was 27 (range 21–36), 5 patients were smokers with pulmonary obstructive disease, and 4 had diabetes mellitus. The median stoma age at surgery was 48 months (range 12–251 months), 3 had a prophylactic mesh at index surgery, 8 patients (31%) had a recurrent PH, where four were mesh repairs and three had a second recurrence. The primary cause for stoma was rectal cancer (13 patients). Other causes were anal or urinary incontinence (three with spinal cord damage), anal fistula, cystitis, constipation, hyperganglionosis, diverticulitis, and ulcerative colitis. The stomas were all end-ostomies distributed as 20 colostomies, 5 ileostomies and 1 urostomy. Six patients had repair of a concomitant hernia with one bilateral r‑TAR and five robotic eRS (Rives–Stoppa, retrorectus) repairs. Three patients had revision of their stomas of which one was unplanned. Serosa lesions occurred in 7 patients and in one a full-wall lesion at skin level led to revision of the stoma. The duration of the procedures varied, with a median procedure time of 156 min (range 107–233 min) for r‑Pauli only and 265 min (range 160–3147 min) for r‑Pauli with additional hernia repair and/or additional stoma revision.

Postoperative complications occurred in eight patients (31%). One patient had stoma necrosis and subcutaneous infection of the stoma bowel, which 3 weeks after the hernia repair was removed and the stoma relocated. Three patients suffered ileus. Three patients had acute pain and one had a second look laparoscopy because of pain without pathology. Flank hematoma or seroma was seen in 4 patients with spontaneous remission in 3 and 1 was drained for fear of infection with negative cultures. Except for the patient with stoma necrosis, no infectious or cardiovascular complications occurred. No patients have reported chronic pain. The median duration of postoperative stay was 3 days (range 1–13 days). One patient with ileus and renal failure and 1 patient with seroma was readmitted. The recurrence rate at the median 14-month follow-up is 3.8% (1/26): The 1 patient with necrosis of the stoma now has a hernia at the previous stoma site. Early necrosis happened because of damage to the vascular supply when freeing the hernia. Possibly this could have been avoided using immunofluorescence (as described above) and/or resection of the devascularized bowel loop.

## The IPST funnel-shaped mesh technique

**Fig. 3:**
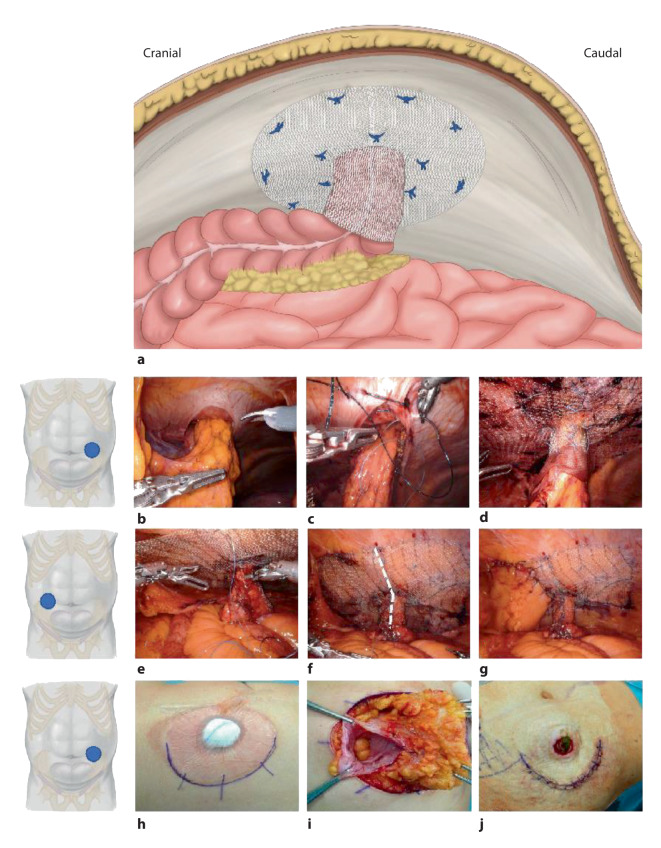
Parastomal hernia repair with funnel-shaped IPST mesh. **a** Overview. **b–d** Repair of a colostomy: **b** clearance of the bowel out of the hernia sac; **c** narrowing of the hernia orifice with suture; **d** final aspect of the IPST mesh repair (in this case the bowel is being protected from the slit edge of the mesh with a piece of Vicryl mesh). **e–g** Repair of an ileostomy: **e** the mesh is placed around the bowel, the funnel part is slit to allow the accommodation of the mesh around the bowel; **f** the slit is suture-closed (*white dotted line*); **g** final aspect with covering of the caudal part of the mesh with peritoneum (partial extraperitonealization of the intraperitoneal onlay mesh [IPOM] mesh). **h–j** Hybrid access to the subcutaneous hernia sac via lateral semicircular peristomal approach: **h** planning of the incision; **i** mobilization of the entrapped bowel and sac; **j** final aspect after positioning of the mesh and reinsertion of the colostomy. (Fig. 3a is adapted from Dietz et al. [21])

A funnel-shaped mesh was developed to combine the properties of funnel effect (to prevent telescoping and prolapse) as well as a narrow collar around the bowel to prevent a PH recurrence. The IPST mesh consists of pure PVDF (polyvinylidene fluoride) on the visceral side and polypropylene/PVDF on the parietal side and has iron particles in the polymeric structure, enabling eventual magnetic resonance imaging if needed (Dynamesh, Aachen, Germany). The funnel is 4 cm in length and has a diameter of 2.5 cm; since it is knitted and has plasticity, the diameter may be digitally enlarged to better accommodate the bowel. It is placed in IPOM position, both prophylactically and in treating PHs ([29]; Fig. 3a; Video 3). There are IPST meshes already slitted and with no slit available.

### Technique

The trocar positioning is performed as described above. After adhesiolysis and reduction of the hernia, the slit IPST mesh is inserted via the 12 mm trocar, placed around the stomal loop, and the funnel is closed using interrupted nonabsorbable sutures (Fig. 3e,f). Afterwards, the flat surface of the mesh is positioned against the abdominal wall, and its slit is closed with sutures (Fig. 3e,f). As described above for the modified Sugarbaker technique, in some circumstances it is reasonable to parietalize partly the groin region to allow a secure mesh placement and ingrowth. This peritoneal flap may cover part of the mesh, reducing the risk adhesions in the future (Fig. 3g). The stoma orifice can be tailored to its ideal diameter using sutures (Fig. 3b,c/colostomy). The mesh fixation is performed using nonabsorbable sutures, in a double-crown technique (Fig. 3a,d).

In cases where the hernia sac, colon or small bowel, mesentery, and omentum are firmly adherent and difficult to dissect from an intra-abdominal approach, high-risk adhesiolysis can be performed in a hybrid procedure, using a peristomal incision (Fig. 3h–j). If the stoma is freed from the skin circumferentially, the mesh may be positioned without the need of slitting it, omitting the step of deployment and fixation during a second endoscopic phase. Before redocking, the stoma orifice is trimmed with nonabsorbable sutures, until the stoma passage is constricted to the appropriate width at the musculoaponeurotic level [10].

### Comments

We have performed this procedure robotically 6 times: 1 for PH of an ileal conduit, 2 for ileostomies (after proctocolectomy), and 3 for end-colostomies. The mean operating time was 201 min (range 129–241 min).

One of the patients with ileostomy and BMI 32 needed a revision on day 5 postoperatively due to ileus. Despite having no pathological findings at the revision, we decided to open the funnel slit (converting the procedure into a keyhole mesh); eventually the patient developed a recurrence due to the keyhole morphology and was successfully treated with a robotic modified Sugarbaker repair (Fig. 1c). In 3 of 6 patients, a hybrid approach with subcutaneous peristomal adhesiolysis was performed. Besides these 6 robotic cases, the authors have an experience of over 100 cases of nonrobotic IPST mesh implantation for prevention and repair of PHs [30].

The 4 cm tunnel length is chosen as the new standard [31] because the stretching of the tunnel with passed bowel with attached mesentary already results in a reduction of the tunnel length. The newly sutured stoma remains in the same position (“in situ” relocation). The IPST technique offers several advantages. The procedure is highly standardized, allows a safe coverage of the vulnerable stoma margins by the elastically of the stretchable funnel, concomitant incisional hernias are treated, there is a low tendency of prolapse and almost no lateral weakness analogous to the modified Sugarbaker technique. Finally, the funnel configuration, with bowel passing through the abdominal wall at a 90° angle, facilitates the irrigation of the stoma. This may be impaired by the lateralization of the bowel after modified Sugarbaker and Pauli procedures.

## Approach to ileal conduit parastomal hernia repair

**Fig. 4:**
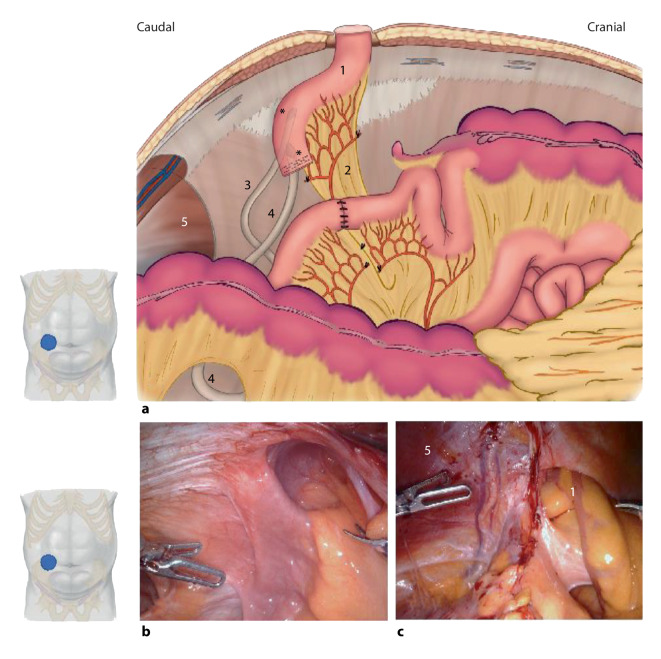
**a** Morphology of an ileal conduit. **b** Complex adhesions to the ileal conduit. **c** Deperitonealized posterior abdominal wall after cystectomy. *1* ileal conduit; *2* mesenterium of the ileal conduit; *3* right ureter; *4* left ureter; *5* deperitonealized lower abdominal wall, *asterisk* uretero-ileal anastomoses

When compared to ileo- or colostomies, ileal conduit PHs pose some specific challenges that complicate their repair (Video 4). Generally, the applied techniques for the surgical treatment of ileal conduit PHs are similar than those used for other PHs. However, depending of the intraoperative findings, this condition requires a tailored approach. This implies a broad surgical armamentarium, in which robotic-assisted techniques are paramount. The specific challenges in the treatment of ileal conduit PHs have been described in a technical note that has recently been published and are summarized below [32]. First, a radical cystectomy often involves removal of the peritoneum and preperitoneal fat below the arcuate line. This sometimes makes an extraperitoneal repair of a PH impossible. In that case, intraperitoneal Sugarbaker or keyhole repairs are preferable. This also complicates closure of the posterior layer in case of transversus abdominis release (TAR) in the treatment of (concomitant) midline incisional hernias. In that case, bridging techniques using omentum or absorbable mesh are sometimes required. Second, the longstanding collapse of an ileal conduit and the presence of ureteric anastomoses often make the dissection of the stomal loop difficult, and prone to complications. The specific anatomical situation after a radical cystectomy and ileal conduit urinary diversion is depicted in Fig. 4. Another observation that is frequently seen in ileal conduit PHs is the difficult lateralization of the stomal loop. Due to an often short mesentery, intraperitoneal or retromuscular modified Sugarbaker repairs may provide insufficient overlap. In that case, keyhole techniques or the use of a preshaped IPST mesh is preferable.

### Tailored approach

In almost half of the patients presenting with an ileal conduit PH, a concomitant midline incisional hernia is present [33]. Despite the fact that minimally invasive techniques are increasingly being used to perform a radical cystectomy, the majority of cases are still performed by open surgery [34]. Along with the challenges described above, this stresses the need for a highly tailored approach in treating ileal conduit PHs. In case of a concomitant midline incisional hernia requiring component separation techniques, a robotic-assisted approach offers unique advantages, and should be treated by robotic-assisted transversus abdominis release (r-TAR), and a Pauli PH repair [11]. The perioperative instillation of the ileal conduit with methylene blue stained saline using a Foley catheter may help in both the identification of the stomal loop, and in detecting any perioperative lesions.

**Fig. 5:**
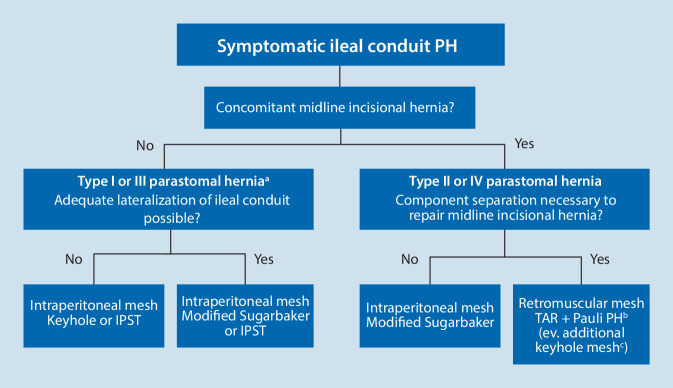
Flow chart of different surgical techniques in ileal conduit parastomal hernia repair [32]. ^a^According to the European Hernia Society classification of parastomal hernias [7]. ^b^Transversus abdominis release (*TAR*) + Pauli procedure with mesh [11]. ^c^In case of inadequate lateralization of the stoma loop, an additional keyhole mesh may be used in the retromuscular plane, with a slit made in the large mesh covering the retromuscular plane. *IPOM* intraperitoneal onlay mesh

The robotic platform allows placement of an extraperitoneal mesh covering all potential hernias. The flowchart presents a possible tailored approach in the robotic-assisted treatment of ileal conduit PHs (Fig. 5).

### Results and comments

Preliminary experience with this standardized approach has recently been published [32]. During a 4-year period, 15 patients underwent a minimally invasive treatment of an ileal conduit PH. Almost half of them (7/15, 46.7%) presented with a concomitant midline incisional hernia. The majority of patients were treated with robotic-assisted laparoscopic surgery (10/15; 66.7%). Median duration of surgery was 197 min, with an interquartile range of 132–260 min. In 33.3% of patients (5/15), the mesh was positioned extraperitoneally, while the remaining patients underwent surgical treatment with an intraperitoneal mesh. In one case robotic-assisted surgery was converted to open due to a perioperative lesion of the stomal loop. Median postoperative hospital stay was 5 days. This series illustrates the high number of complications after the surgical treatment of ileal conduit PHs, with almost half of them (7/15, 46.7%) presenting with any complication within 30 days after surgery. One third of patients developed postoperative urinary infection and 2 patients required intensive care during hospitalization. Median follow-up was 366 days. One patient developed a local recurrence of her PH on day 66 postoperatively, which was treated with intraperitoneal mesh.

Evidence on the surgical treatment of ileal conduit PHs is scarce [11] [33–35]. Until now, only retrospective patient series have been published, including limited numbers of patients. Recently, a nation-wide analysis from Finland illustrated the substantial morbidity postsurgery [26]. They retrospectively analyzed outcomes in 28 patients treated between 2007 and 2017. During a median follow-up of 30 months, 18% of recurrences and 14% of complications were noted. Slightly better outcomes of the modified Sugarbaker technique were reported, compared to keyhole repairs. In general, this finding was not confirmed in any other patient cohort, and available literature does not allow to determine the optimal local treatment of ileal conduit PHs. Novel developments, like robot-assisted techniques, treatment of concomitant midline incisional hernias using r‑TAR or PH hernia repair using the Pauli technique are not included in any available cohort data. These observations illustrate the complexity in treating this specific condition, and stress the need for additional evidence. Finally, although there is consensus that adapting the type of repair to the individual characteristics of the patient and the findings of the ileal conduit PH is paramount, tailoring will probably also delay even more the maturation of evidence in specialized centers. Large registry data will probably be needed.

## Conclusion

- Parastomal hernias significantly impair quality of life.
- The surgical treatment of parastomal hernias is complex, and prone to complications and recurrences.
- Several techniques for the local repair of parastomal hernias have been proposed; however, current evidence does not allow the identification of the optimal surgical technique.
- Surgery for the treatment of parastomal hernias is increasingly being performed using minimally invasive techniques. Robot-assisted surgery offers specific advantages, like avoiding penetrating fixation techniques, easy suture closure of the hernia defect, the facilitation of extraperitoneal mesh placement, and the implementation of advanced component separation techniques.
- The optimal surgical technique in parastomal hernia repair should be tailored to the patient’s history, characteristics of the parastomal hernia, and the presence of a concomitant midline incisional hernia.

## Supplementary Information

Video 1: Modified Sugarbaker technique.

Video 2: Pauli technique.

Video 3: IPST technique.

Video 4: Treatment of a parastomal hernia at an ileum conduit

## Declarations

The data collection of this study regarding the IPST mesh (Kantonsspital Olten) is based on a positive ethics vote of the Ethics Committee Northwestern Switzerland (No. 2019-02046), and the study was performed based on the ethical guidelines specified therein. For the remaining data presented in this manuscript, the ethics approval is disclosed in the respective original publications (see references). For images or other information in the manuscript that can be used to identify patients, written consent was obtained from the patients and/or their legal representatives.
